# Supplementary material for: Canonical PRC1 controls sequence-independent propagation of Polycomb-mediated gene silencing
Source: Nat Commun. 2019 Apr 29;10:1931. doi: 10.1038/s41467-019-09628-6 (PMC6488670; doi:10.1038/s41467-019-09628-6)
Supplement: Supplementary file 1 — Supplementary Information [file 41467_2019_9628_MOESM1_ESM.pdf]

Canonical PRC1 controls sequence-independent propagation of Polycomb-dependent gene silencing

Moussa et al.

Supplementary Figure 1

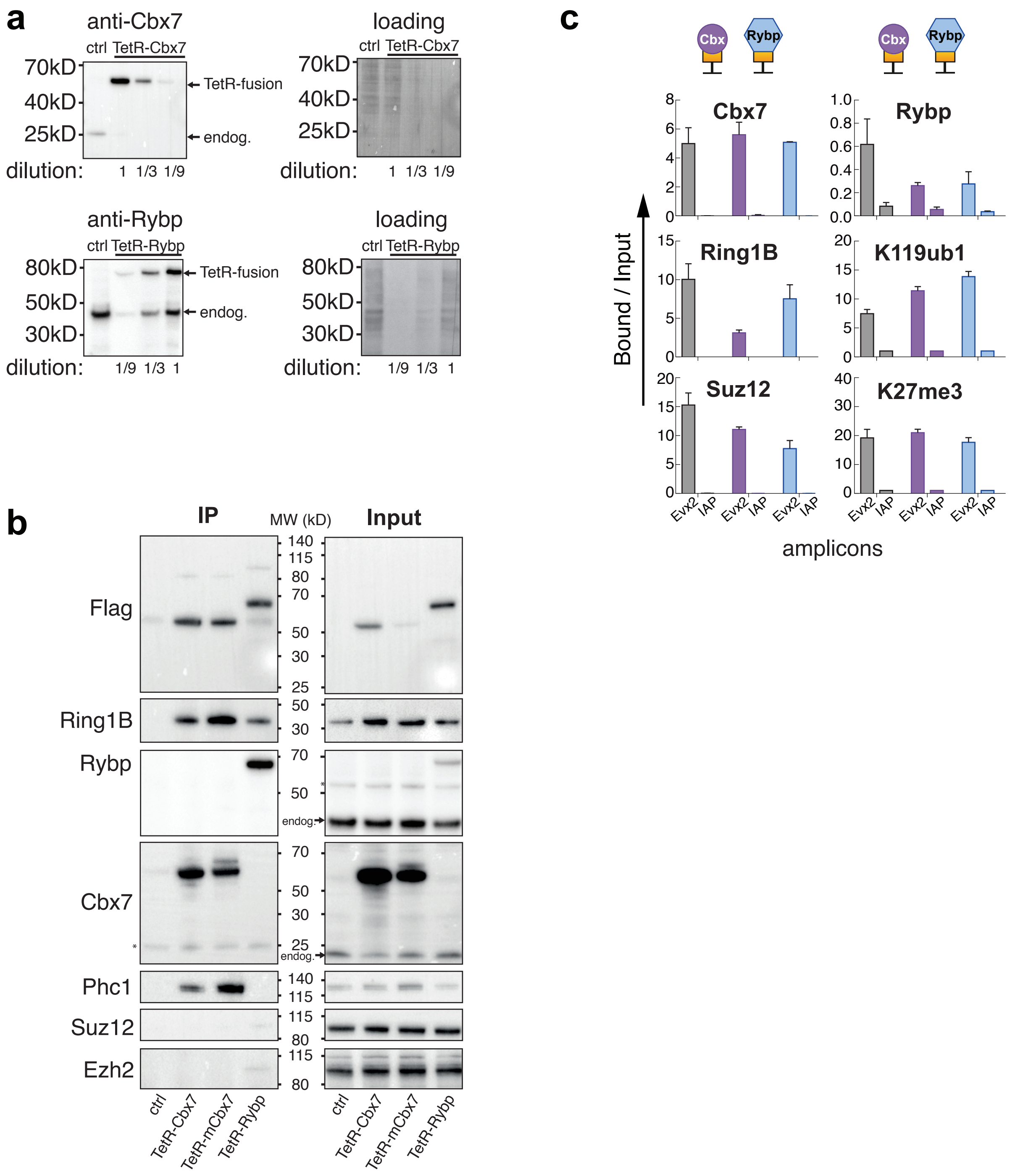

Supplementary Figure 1. Ectopic expression of TetR fusion proteins results in selective association with endogenous PRC1 components and does not disrupt normal Polycomb regulation and cell proliferation. a Immuno blots showing expression levels of TetR fusion proteins relative to endogenous Cbx7 and Rybp. Ponceau membrane staining serves as loading controls. **b** Co-immunoprecipitation analysis of FLAG-epitope-tagged TetR fusions with Cbx7, mutant mCbx7 (TetR-Cbx7W35A) and Rybp in TetO-mESCs. PRC1 core subunit Ring1B, but not PRC2 components Suz12 and Ezh2, co-purify with TetR fusions of Cbx7, mCbx7 and Rybp. Canonical PRC1 subunit Phc1 associates specifically with TetR-Cbx7 and TetR-mCbx7. Parental TetO-mESCs serve as negative control. **c** ChIP qPCR analyses of PcG proteins and histone modifications in the absence and presence of TetR fusion proteins at the Evx2 promoter (positive control) and at IAP (negative control). ChIP enrichments for H2AK119ub1 and H3K27me3 are normalized to IAP. Data are mean ±SD (error bars) of three independent experiments.

Supplementary Figure 2

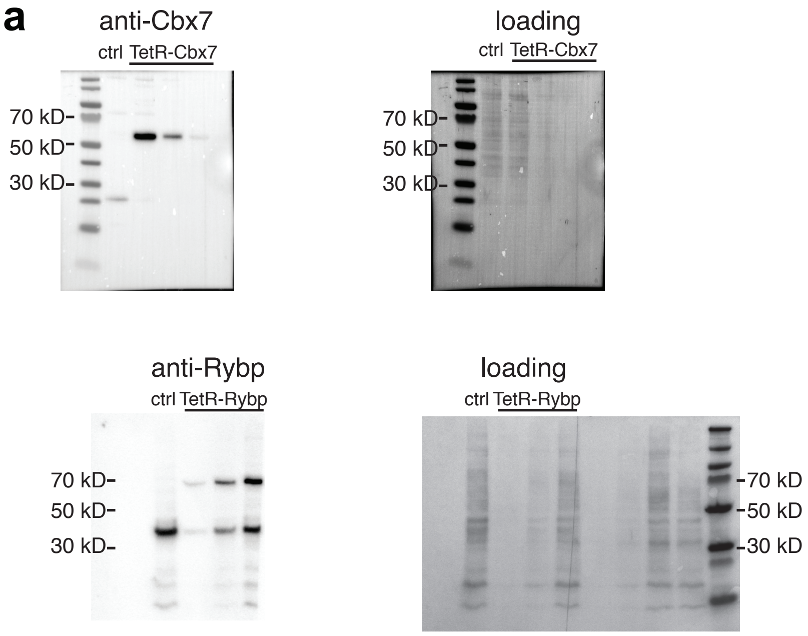

Supplementary Figure 2. Uncropped western blots of proteins from Supplementary Figure 1a a) Western blots of Cbx7 and Rybp in TetO-mESCs and cPRC1-TetO- and vPRC1-TetO-mESCs. Antibody names are noted above blots, numbers on the side represent mass in kD based on the protein marker. Coomassie staining indicates loading.

Supplementary Figure 3

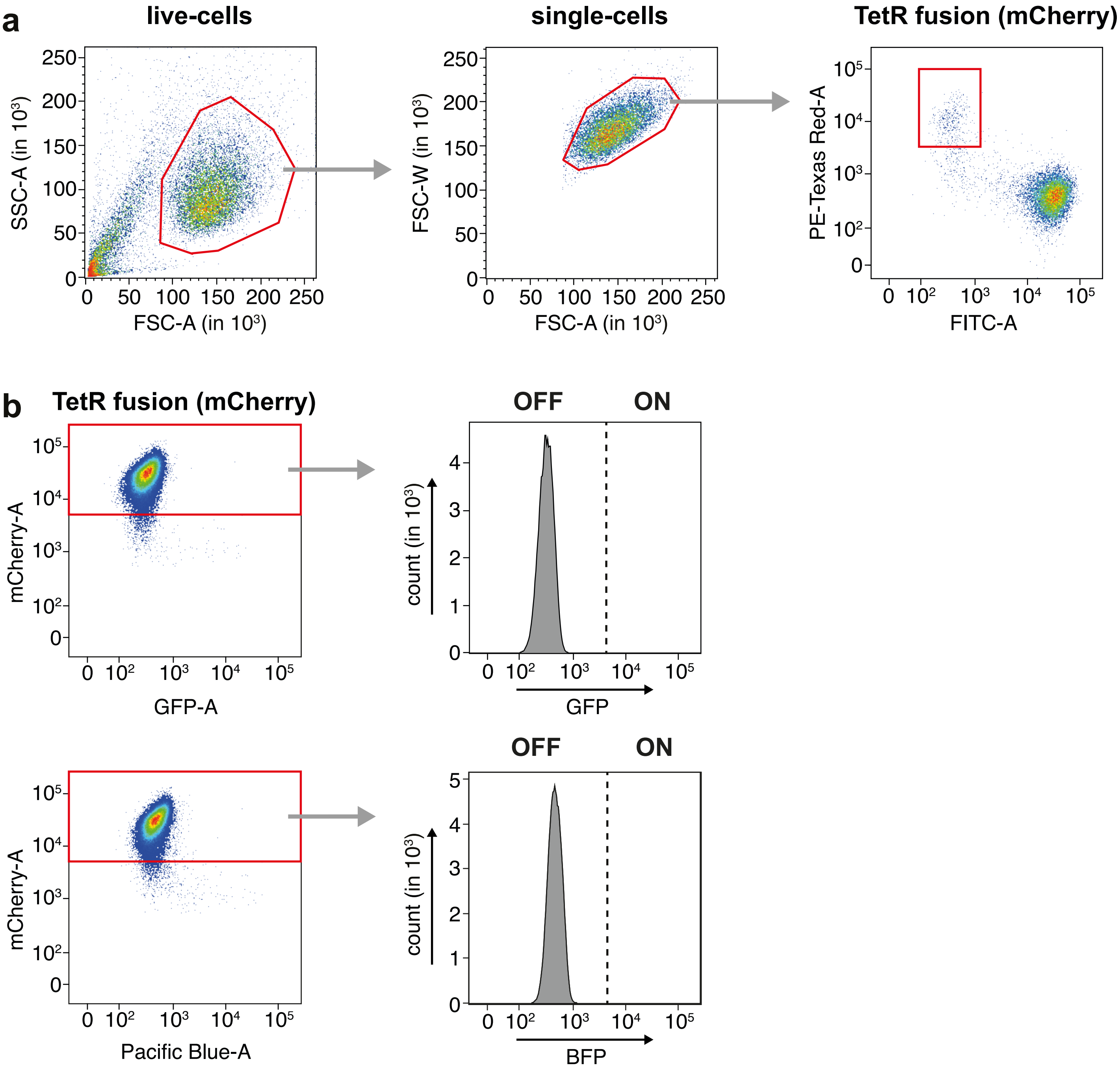

Supplementary Figure 3. Flow cytometry-based isolation and analyses of wild-type and CRISPR mutant cPRC1- and vPRC1-TetO-mESCs. a) Strategy to isolate single cell clones of cPRC1-TetO- and vPRC-TetO-mESCs. All mESC clones expressing either TetR-Cbx7 (Figures 1, 2, 3, 4), TetR-Rybp (Figures 1, 2, 3, 4), TetR-Cbx7^{W35A} (Figures 4) as well as loxp flanked TetR fusions (Figure 2e, f, Supplementary Figure 7) were isolated by FACS gated for "mCherry-positive", "live-cells" and "single-cells"; independent of GFP or BFP expression. **b)** Flow cytometry histograms for Figures 1d, 2a-f, 3a, 3b, 4b-d were generated from "mCherry-positive" TetO-mESCs.

Supplementary Figure 4

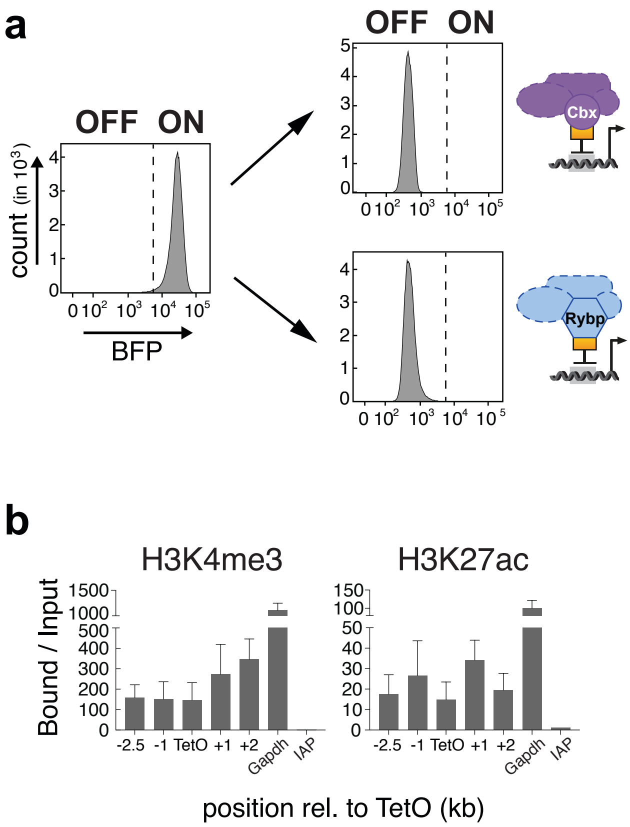

Supplementary Figure 4. Active histone modifications decorate the 7xTetO site and flanking tran-scriptionally active reporter genes in parental TetO-mESCs. a Flow cytometry histograms of BFP expression in the absence and presence of TetR PcG fusion proteins. **b** ChIP analyses of active histone modifications in the absence of TetR fusion proteins. Gapdh and IAP serve as positive and negative controls, respectively. ChIP enrichments are normalized to IAP. Data are mean ±SD (error bars) of three independent experiments. **c** Flow cytometry histogram of GFP expression in TetO-mESCs expressing TetR DNA binding domain without fusion partner.

Supplementary Figure 5

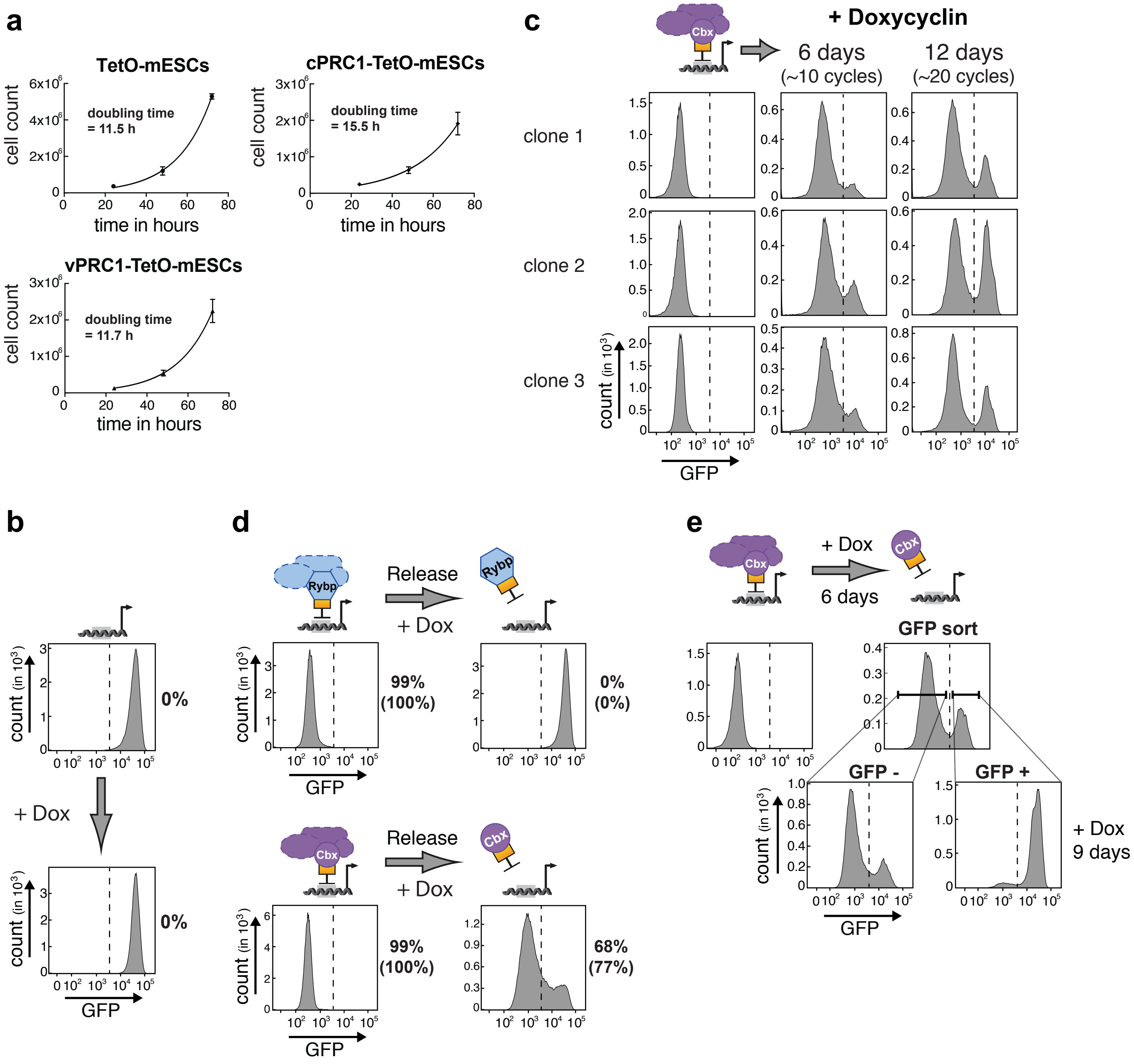

Supplementary Figure 5. Release of TetR-Cbx7 creates a bimodal cell population that persists through DNA replication and cell division. a Growth curves of parental TetO-mESCs, cPRC1-TetO- and vPRC1-TetO-mESCs. Doubling times were determined based on two independent cell count measurements in 24 hours intervals as indicated. **b** Flow cytometry histograms of GFP expression in parental TetO-mESCs before and after six days of Dox treatment. **c** Flow cytometry histogram of GFP expression before and after Dox treatment of three different clonal populations of cPRC1-TetO-mESCs. **d** Flow cytometry histograms of GFP expression of parental TetO-ESC populations transduced with TetR-Cbx7 or TetR-Rybp before and after six days of Dox treatment. **e** Flow cytometry histogram of cPRC1-TetO-mESCs treated with Dox for additional three days after FACS of GFP-positive and –negative cells in response to TetR-Cbx7 reversal.

Supplementary Figure 6

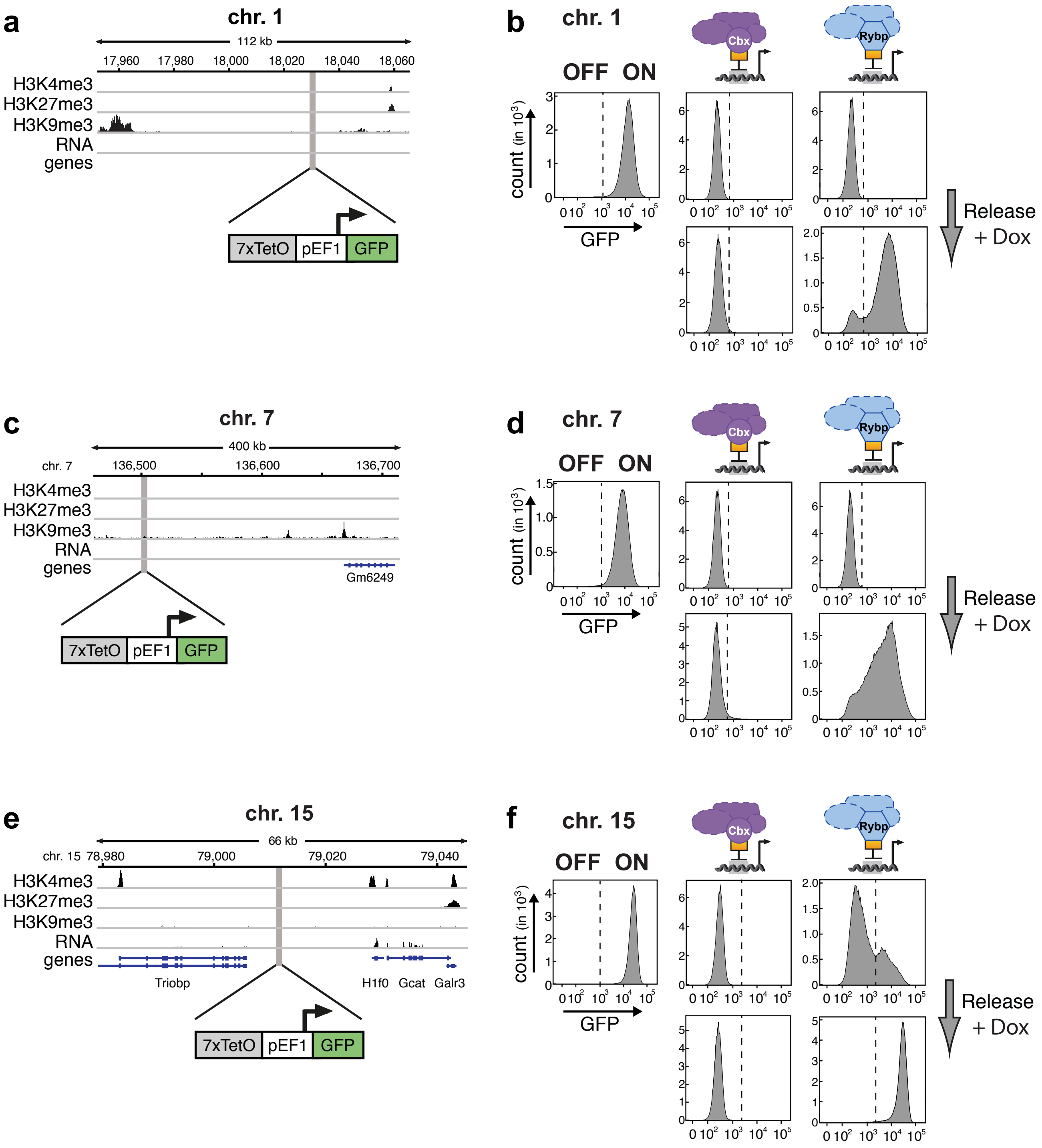

Supplementary Figure 6. Differential maintenance by cPRC1 and vPRC1 is independent of TetO reporter and chromosomal insertion site. a, **c** and **e** Histone modifications and RNA expression surrounding the integration site of a single reporter gene construct located either on chromosomes 1, 7 or 15 in three independent TetO-mESCs. **b, d** and **f** Flow cytometry histograms relate GFP expression in the absence of TetR PcG fusion proteins, and before and after reversal of TetR fusion protein recruitment in response to Dox treatment for six days.

Supplementary Figure 7

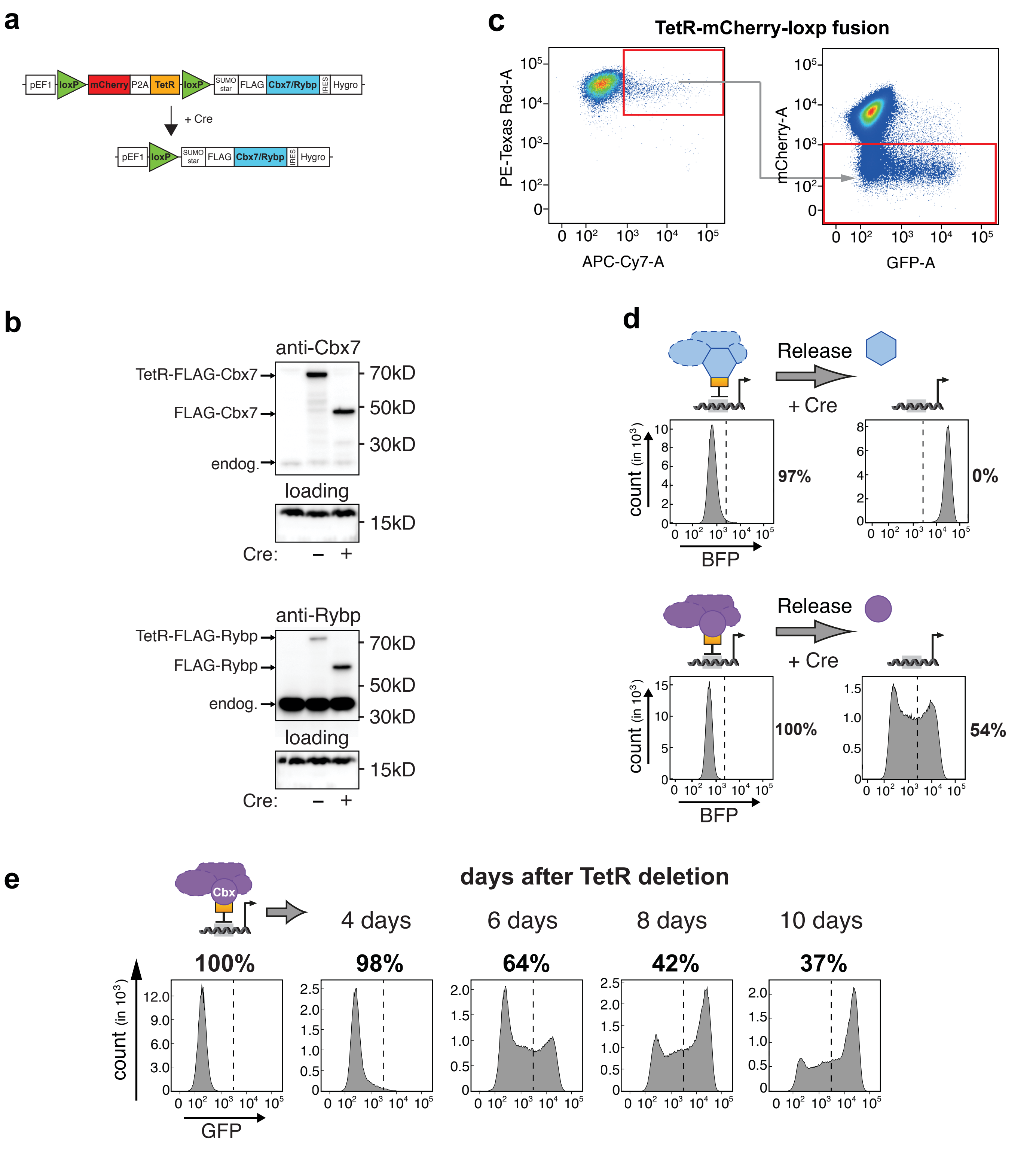

Supplementary Figure 7. Genetic release of TetR binding reveals specific heritable gene silencing by cPRC1. a Schematic representation of TetR fusion transgenes that enable genetic release of TetR bind- ing to validate heritable maintenance of chromatin modifications and expression state in the absence of the initial stimulus. The DNA sequences encoding mCherry and the TetR DNA binding domain are flanked by two loxp sites enabling Cre recombinase mediated deletion without disrupting downstream DNA sequences. Horizontal black bars indicate primer binding sites for PCR genotyping. **b** Immuno blot of Cbx7 and Rybp protein expression before and after transfection with Cre recombinase in TetO-mESCs with transgenes harboring conditional TetR DNA binding domain. Membrane staining with Coomassie serve as loading controls. **c** “mCherry-negative” cPRC1-TetO- and vPRC-TetO-mESCs, which lack the TetR DNA binding domain, were analyzed after gating for successful Cre-recombinase transfection (APC-Cy7). **d** Flow cytometry histograms of BFP expression before and after genetic release of TetR DNA binding six days after transfection with Cre recombinase. **e** Flow cytometry histo- grams showing time-course of GFP repression before and after Cre-mediated TetR deletion in cPRC1-mESCs.

Supplementary Figure 8

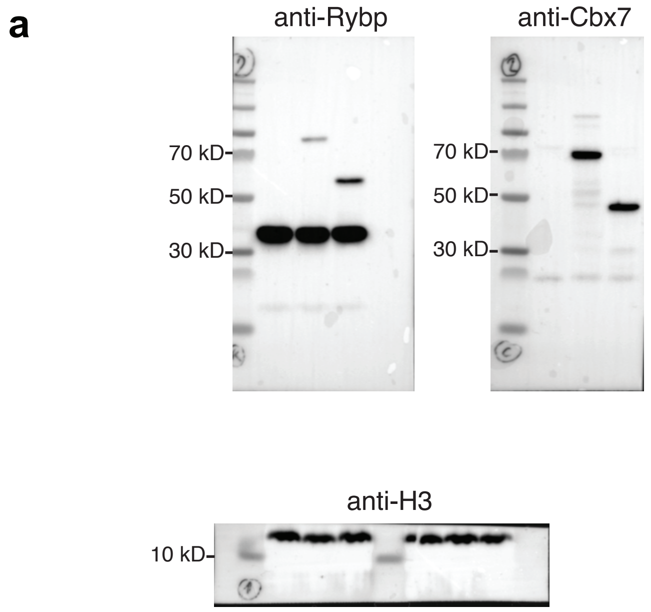

Supplementary Figure 8. Uncropped western blots of proteins from Supplementary Figure 7b
a) Western blots of Rybp and Cbx7 before and after Cre-mediated excision of TetR DNA binding domain. Antibody names are noted above blots, numbers on the side represent mass in kD based on the protein marker. H3 was used for loading.

Supplementary Figure 9

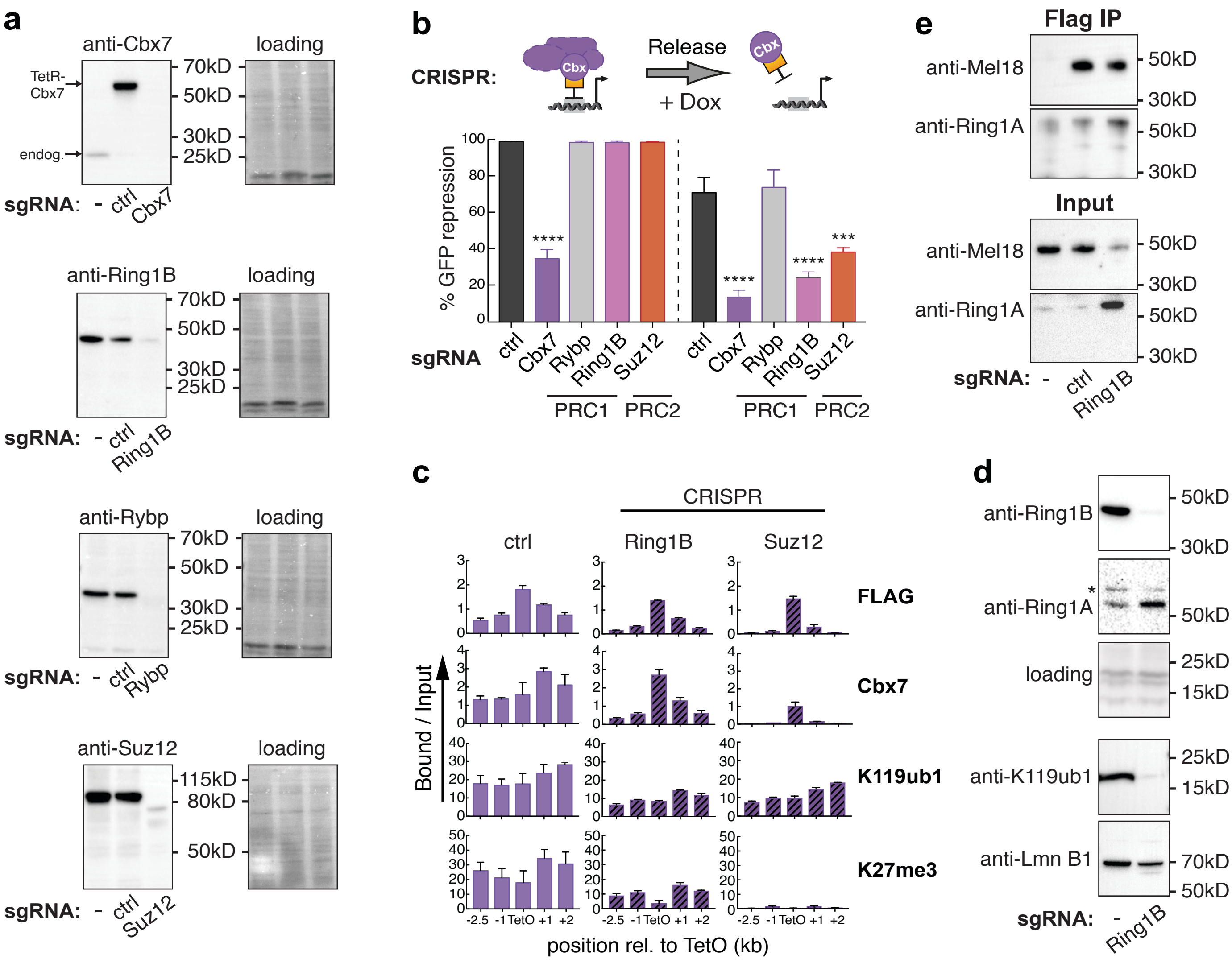

Supplementary Figure 9. Functional canonical PRC1 and PRC2 but not variant PRC1 are required to maintain gene silencing established by cPRC1. a Immuno blots of cPRC1-TetO-mESCs express-ing Cas9 together with sgRNAs targeting endogenous PcG proteins. sgRNA against Slc6a6 serves as control. Membrane staining with Coomassie serves as loading control. **b** Quantification of flow cytometry analyses of cPRC1-TetO-mESCs expressing Cas9 and sgRNAs against endogenous PRC1 and PRC2 components from at least three independent experiments. Bar graph shows mean and standard devia-tion of the percentage of GFP-negative cells before and after six days of Dox. Statistical significance was calculated relative to controls (ctrl) using Student t-test (P values: ≥ 0.05; ≤ 0,05 (*); ≤ 0,01 (**); ≤ 0,001 (***); ≤ 0,0001 (****)). **c** ChIP-qPCR analysis compares the relative enrichments of TetR fusion, Cbx7 proteins and histone modifications in cPRC1-TetO-mESCs and CRISPR mutant clones of cPRC1-Te- tO-mESCs with Ring1B LOF and Suz12 LOF. ChIP enrichments for H2AK119ub1 and H3K27me3 are normalized to IAP. Data are mean ±SD (error bars) of at least two independent experiments. Source data are provided as a Source Data file. **d** Immuno blot shows Ring1A expression and bulk levels of H2AK119ub1 in Ring1B CRISPR mutant clone. Lmn B1 and membrane staining with Coomassie serve as loading controls. **e** Immuno blots showing co-Immunoprecipitation of Mel18 and Ring1A in wild-type, cPRC1-TetO mESCs and cPRC1-TetO Ring1B CRISPR mutants mESCs.

Supplementary Figure 10

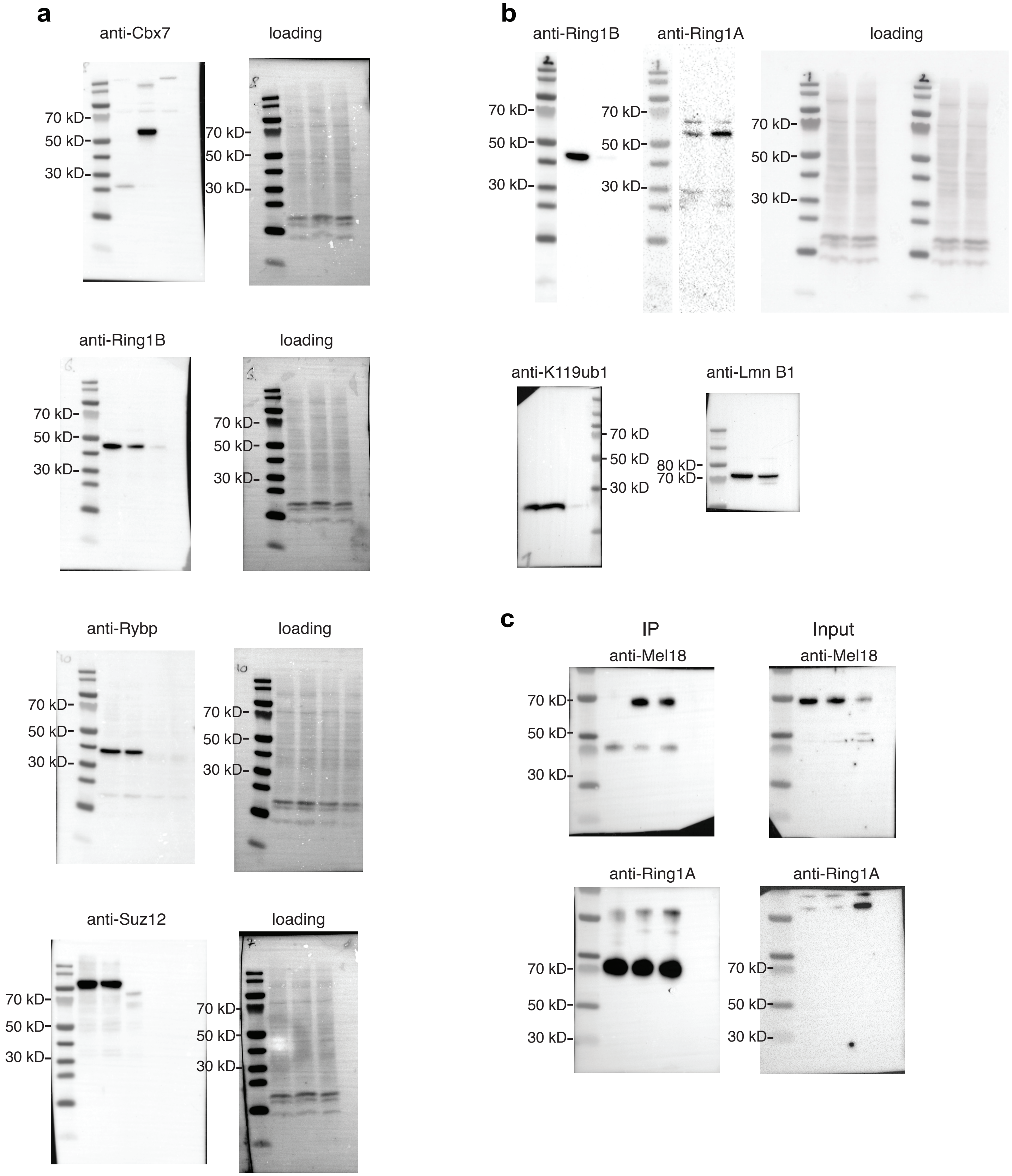

Supplementary Figure 10. Uncropped Western blots of proteins from Supplementary Figure 9 a), d) and e). a) Western blots of Supplementary Figure 9 a) showing CRISPR-mediated loss-of-function. **b)** Western blots of Supplementary Figure 9 d). **c)** Western blots of Supplementary Figure 9 e). Antibody names are noted above blots, numbers on the side represent mass in kD based on the protein marker.

Supplementary Figure 11

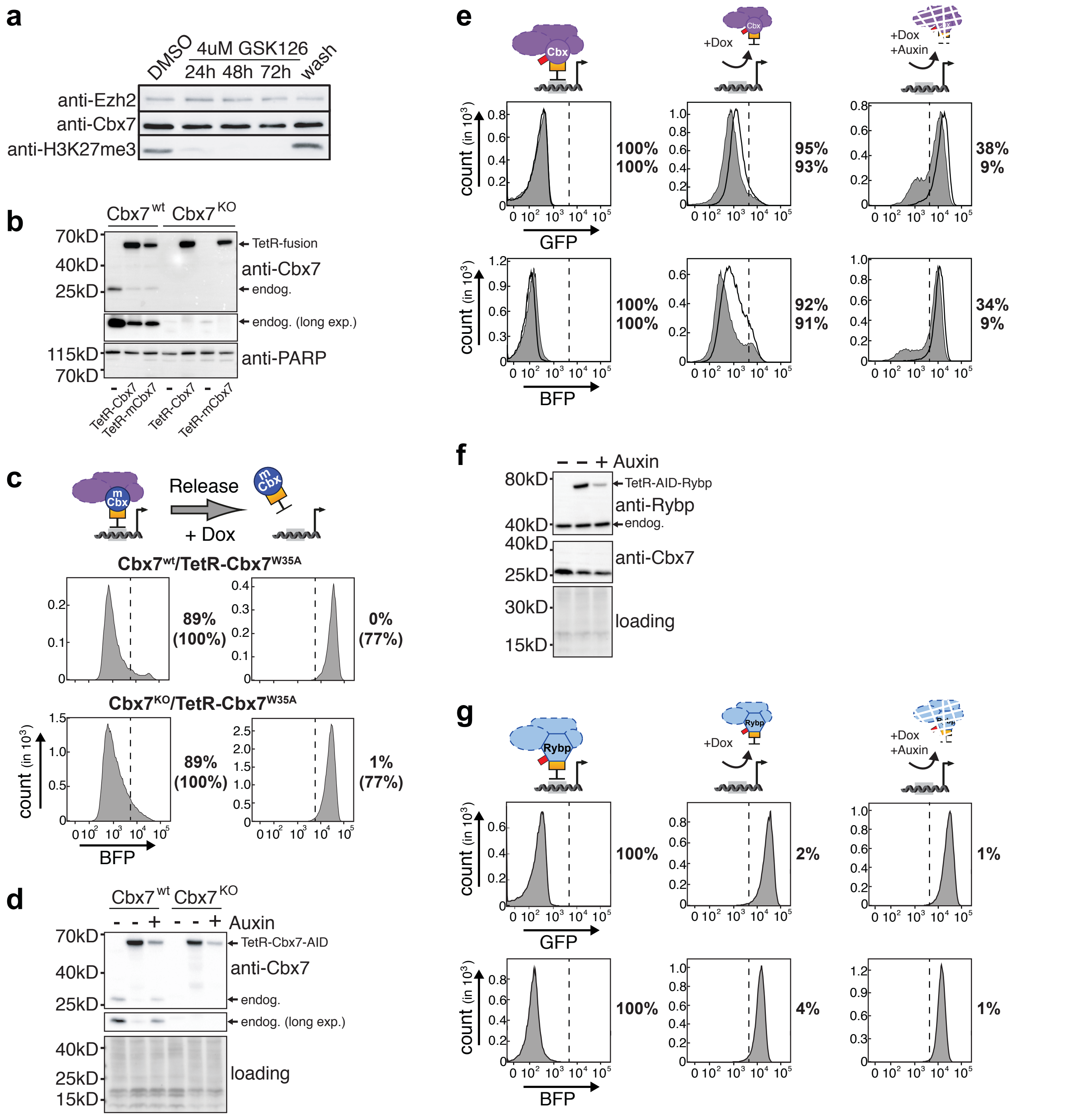

Supplementary Figure 11. Auxin-inducible degradation reveals contribution of endogenous Cbx7 to cPRC1-specific propagation of heritable gene silencing. a Immuno blots of H3K27me3, Ezh2 and Cbx7 show specific and reversible inhibition of the catalytic activity of PRC2 after treatment of parental TetO-mESCs with 4uM of GSK126 without disrupting endogenous PRC2 and cPRC1 stability. **b** Immuno blot shows impact of ectopic expression of TetR-Cbx7 and mutant TetR-mCbx7 (TetR-Cbx7W35A) on endogenous Cbx7 levels and confirms generation of LOF mutation in endogenous Cbx7 gene (Cbx7KO). Detection of PARP serves as a loading control. **c** Flow cytometry histograms compare BFP expression before and after three days of Dox treatment of wild-type (gray - upper panels) and Cbx7KO TetO-mESCs expressing TetR-Cbx7W35A (gray - lower panels). Percentages indicate fraction of silenced reporter cells expressing either mutant or wild-type TetR fusion (in brackets). **d** Immuno blot shows expression of TetR-Cbx7-AID in wild-type and Cbx7KO TetO-mESCs. 72 hours of Auxin treatment induces degradation of the TetR-fusion protein and results in upreg- ulation of endogenous Cbx7 in wild-type cPRC1-mESCs. Membrane staining with Coomassie serves as load- ing control. **e** GFP and BFP histograms of wild-type (grey) and Cbx7KO reporter cells (no fill) expressing TetR-Cbx7-AID. Flow cytometry analysis shows before and after three days of treatment with Dox alone or Auxin and Dox combined. Percentages indicate fraction of silenced cells in wild-type (top) and Cbx7KO report- er cells (bottom). **f** Immuno blot shows expression of TetR-AID-Rybp in wild-type TetO-mESCs. 72 hours of Auxin treatment induces degradation of the TetR-fusion protein. Endogenous expression levels of Rybp and Cbx7 remained unaffected. Membrane staining with Coomassie serves as loading control. **g** GFP and BFP histograms of TetO-mESCs expressing TetR-AID-Rybp. Flow cytometry analysis shows before and after three days of treatment with Dox alone or Auxin and Dox combined. Percentages indicate fraction of silenced cells.

Supplementary Figure 12

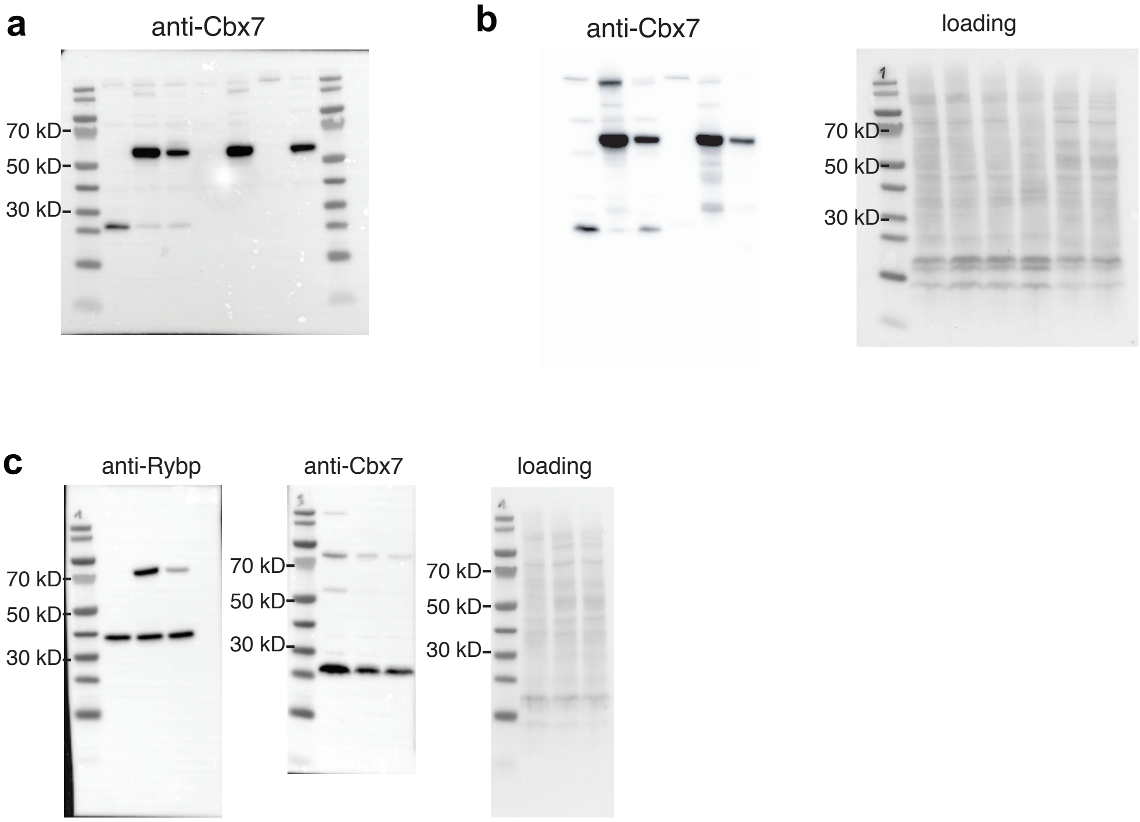

Supplementary Figure 12. Uncropped Western blots of proteins from Supplementary Figure 11 b), **d)** and **f). a)** Western blots of Supplementary Figure 11 b). **b)** Western blots of Supplementary Figure 11 d). **c)** Western blots of Supplementary Figure 11 f). Antibody names are noted above blots, numbers on the side represent mass in kD based on the protein marker.

Supplementary Figure 13

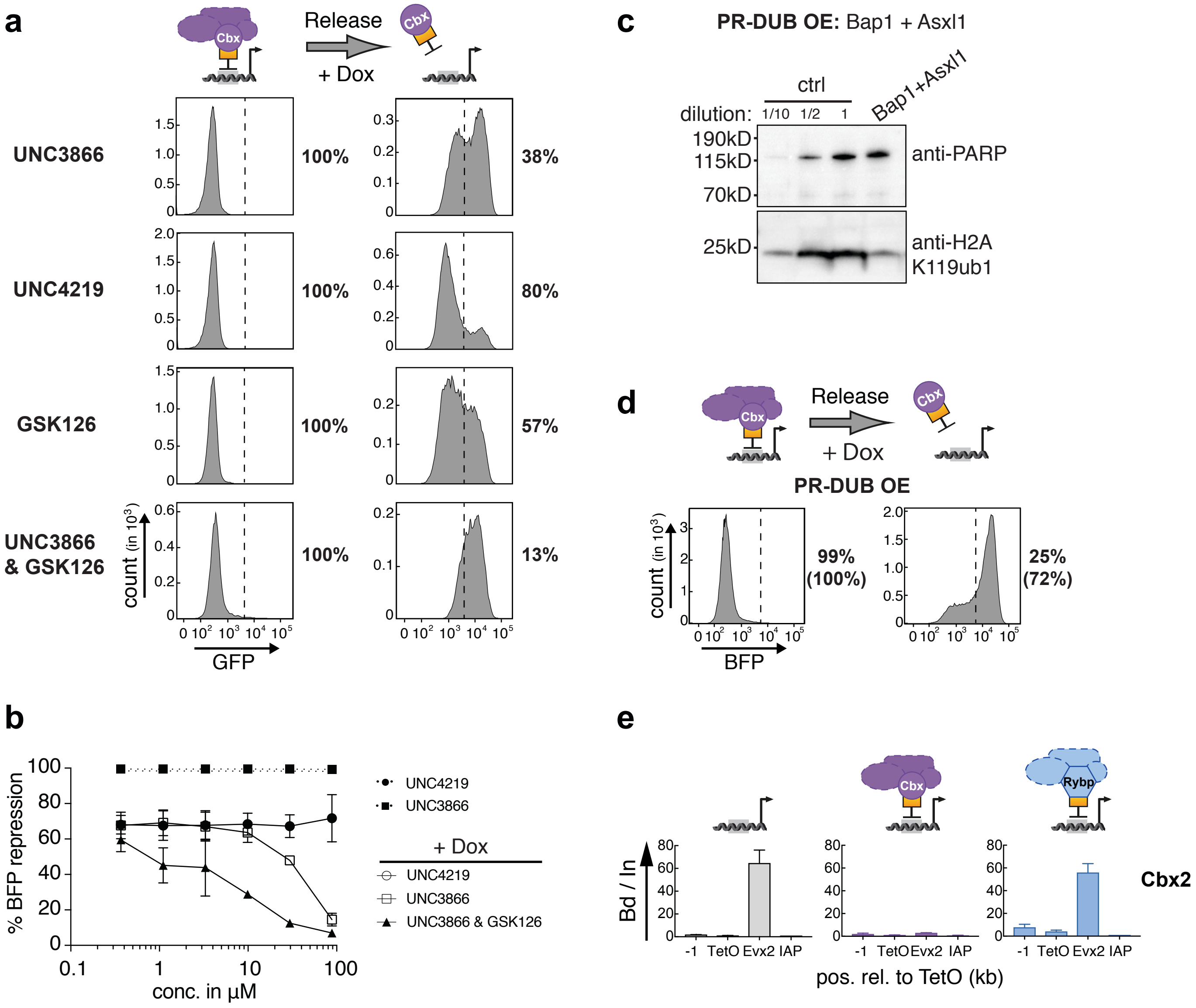

Supplementary Figure 13. Feedback interactions with H3K27me3 and H2AK119ub1 are critical for heritable gene silencing by canonical PRC1. a Representative flow cytometry analyses of GFP expression used in Figure 3b in the context of 30uM of UNC3866, 30uM of UNC4219, 4uM GSK126 or a combination of 30uM of UNC3866 and 4uM GSK126. **b** Percentage of BFP-negative cells before and after six days of Dox treatment in response to increasing concentrations of Cbx7 inhibitor (UNC3866) alone, in combination with 4uM GSK126 or the control compound (UNC4219). **c** Immuno blot compares bulk level of H2AK119ub1 in cPRC1-TetO-mESCs at different dilutions with H2AK119ub1 levels in cPRC1-TetO-mESCs expressing ectopic Bap1 and N-terminal Asxl1 (1–479 aa). Detection of PARP serves as a loading control. **d** BFP histograms before and after six days of Dox addition to cPRC1-TetO-mESCs without and with overexpression of Bap1 and Asxl1 (PR-DUB OE), components of the human PR-DUB complex specific for H2AK119ub1. Parental cPRC1-TetO-mESCs (no fill) serve as reference. Percentages indicate fraction of silenced cPRC1-TetO-mESCs with PR-DUB OE and without (in brackets). **e** ChIP-qPCR analysis compares the relative enrichments of Cbx2 proteins at TetO site, Evx2 promoter (positive control) and at IAP (negative control) in TetO-mESCs expressing TetR fusions. Similar to endogenous Cbx7, ectopic expression of TetR-Cbx7 leads to downregulation of endogenous Cbx2. Data are mean ±SD (error bars) of three independent experiments.

Supplementary Figure 14

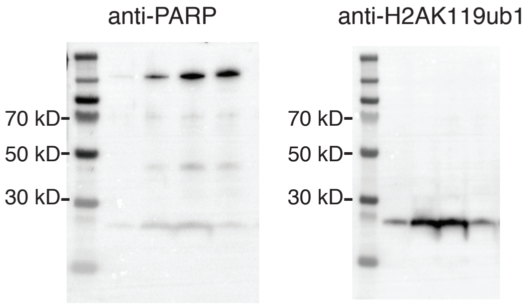

Supplementary Figure 14. Uncropped Western blots of proteins from Supplementary Figure 13 c) Antibody names are noted above blots, numbers on the side represent mass in kD based on the protein marker.

Supplementary Tables

Supplementary Table 1. List of plasmids

Plasmid #	Description
YR06	frt- ZFHD1-GAL4-pEF1a-NLS-tagBFP-pA-loxp-7xTetO-pPGK1-PURO-EGFP-loxp-pA-F3
Db51	nLV_EF1a_TetR_2xGGGS_3xFLAG_Cbx7_W35A_P2A_mCherry
Db55	nLV_UCOE SFFV_Asxl1_P2A_Bap1_P2A_BLAST
HFM91	Chr7_loxP-Ef1a-LoxP-CP039_RMCE_tetO_loxP-Ef1a-LoxP_CpGlessGFP
HFM92	Chr15_loxP-Ef1a-LoxP-CP039_RMCE_tetO_loxP-Ef1a-LoxP_CpGlessGFP
HFM93	Chr1_loxP-Ef1a-LoxP-CP039_RMCE_tetO_loxP-Ef1a-LoxP_CpGlessGFP
Db52	nLV_EF1a_TetR_2xGGGS_3xFLAG_AID_2xGGGS_Cbx7_P2A_mCherry
Db53	nLV_UCOE_SFFV_Tir1_3xMyc_IRES_Hygro_PolyA
Db75	Bluescript SK_pGK-Cre-IRES-Thy1.1
YR111	nLV_EF1a-loxP-mcherry-P2A-TetR-2xGGGS-V5-loxP-FLAG-SUMOtag-Cbx7-IRES-Hygro-PolyA
YR112	nLV_EF1a-loxP-mcherry-P2A-TetR-2xGGGS-V5-loxP-FLAG-SUMOtag-Rybp-IRES-Hygro-PolyA
Db86	nLV_EF1a-Kozak-V5-2xGGGS-NotI-Cbx7-NotI-P2A-BLAST
CP012	nLV_EF1a_TetR_2xGGGS_3xFLAG_Cbx7_P2A_mCherry
CP013	nLV_EF1a_TetR_2xGGGS_3xFLAG_RYBP_P2A_mCherry
CP014	nLV_EF1a_TetR_2xGGGS_3xFLAG_EED_P2A_mCherry
AA008	nLV_Dual_Promoter_EF1a_Gal4_2xGGGS_RYBP_BLAST
AA007	nLV_Dual_Promoter_EF1a_Gal4-2xGGGS_Cbx7_BLAST
N0103	nLV_Dual_Promoter_EF1a _Gal4-2xGGGS _BLAST

Supplementary Table 2. Antibodies

Epitope	Cat #	Vendor	Host	WB
Ring1B	5694S	Cell Signaling	rb	1:1000
Cbx7	07-981	Millipore	rb	1:1000
Rybp	41787S	Cell Signaling	rb	1:1000
Eed	61203	Active Motif	ms	1:500
LaminB1	ab16048	Abcam	rb	1:5000
Ring1A	2820S	Cell Signaling	rb	1:1000
Suz12	3737S	Cell Signaling	rb	1:1000
H3K27me3	pAb-069-050	Diagenode	rb	1:1000
H2AK119ub1	8240S	Cell Signaling	rb	1:20000
FLAG	F1804	Sigma	ms	
Goat anti-Rabbit IgG HRP	656120	Invitrogen	gt	1:10000
anti-Mouse IgG HRP	W4021	Promega	gt	1:2500

Supplementary Tables

Supplementary Table 3. Primers used for ChIP-qPCR

Primer	5'-3' sequence
Evx2_sense	CGCAGCCCATCATTAAGAC
Evx2_antisense	CGGACAAACTGGAGAACCTC
GAPDH_sense	CTCTGCTCCTCCCTGTTCC
GAPDH_antisense	TCCCTAGACCCGTACAGTGC
IAP_sense	CTCCATGTGCTCTGCCTTCC
IAP_antisense	CCCCGTCCCTTTTTTAGGAGA
TetO_DBS_sense	AAGATGGGCTGCAGGAATTC
TetO_DBS_antisense	ATACACGCCTACCTCGACATAC
TetO_+2kb_sense	CAGTGCCACGTTGTGAGTTG
TetO_+2kb_antisense	GCCCCTTGTTGAATACGCTTG
TetO_+1kb_sense	GATCCGGACCGCCACATC
TetO_+1kb_antisense	ACACCTTGCCGATGTCGAG
TetO_-1kb_sense	TCACTTCAAGTGCACATCCG
TetO_-1kb_antisense	TCGACCACCTTGATTCTCATGG
TetO_-2.5kb_sense	GGCTTAATGATGGGCGTTGT
TetO_-2.5kb_antisense	TCCGACTAGTTCAATGACAGGA

Supplementary Table 4. CRISPR gRNA sequences

Target gene	5'-3' CRISPR guide sequence
Suz12 gRNA Exon 7	AGTCCCTACTGGTAAAAAGC
Slc6a6 gRNA Exon 4	GGCCAGTACACATCAGAAGG
Eed gRNA Exon 6	CTCATTGATAGCATTTCCA
Cbx7 gRNA Exon 1	GCATCCGGAAGAAGCGCGTG
Rybp gRNA Exon 2	AATGCAGCATCTGCGATGTG
Ring1B gRNA Exon 4	ATTAATGTGCCCAATTTGTT
Ring1B gRNA	GTGTTTACATCGGTTTTGCG
Suz12 gRNA	ACCTCCATGTCATGAAGCAT
chr1_guide	CTTGAACATAGCAACAGCAC
chr15_guide	ACAGCGCTCTCATTTTACAG
chr7_guide	TGCTCTCTGTATATAAGAAG
