# Supplementary material for: Canonical PRC1 controls sequence-independent propagation of Polycomb-mediated gene silencing
Source: Nat Commun. 2019 Apr 29;10:1931. doi: 10.1038/s41467-019-09628-6 (PMC6488670; doi:10.1038/s41467-019-09628-6)
Supplement: Supplementary file 3 — Reporting Summary [file 41467_2019_9628_MOESM3_ESM.pdf]

nature research

Corresponding author(s): Oliver Bell
Last updated by author(s): Feb 4, 2019

Reporting Summary

Nature Research wishes to improve the reproducibility of the work that we publish. This form provides structure for consistency and transparency in reporting. For further information on Nature Research policies, see Authors & Referees and the Editorial Policy Checklist.

Statistics

For all statistical analyses, confirm that the following items are present in the figure legend, table legend, main text, or Methods section.

n/a	Confirmed	
☐	☒	The exact sample size (*n*) for each experimental group/condition, given as a discrete number and unit of measurement
☐	☒	A statement on whether measurements were taken from distinct samples or whether the same sample was measured repeatedly
☐	☒	The statistical test(s) used AND whether they are one- or two-sided<br>*Only common tests should be described solely by name; describe more complex techniques in the Methods section.*
☒	☐	A description of all covariates tested
☒	☐	A description of any assumptions or corrections, such as tests of normality and adjustment for multiple comparisons
☒	☐	A full description of the statistical parameters including central tendency (e.g. means) or other basic estimates (e.g. regression coefficient) AND variation (e.g. standard deviation) or associated estimates of uncertainty (e.g. confidence intervals)
☐	☒	For null hypothesis testing, the test statistic (e.g. *F*, *t*, *r*) with confidence intervals, effect sizes, degrees of freedom and *P* value noted<br>*Give P values as exact values whenever suitable.*
☒	☐	For Bayesian analysis, information on the choice of priors and Markov chain Monte Carlo settings
☒	☐	For hierarchical and complex designs, identification of the appropriate level for tests and full reporting of outcomes
☒	☐	Estimates of effect sizes (e.g. Cohen's *d*, Pearson's *r*), indicating how they were calculated

Our web collection on statistics for biologists contains articles on many of the points above.

Software and code

Policy information about availability of computer code

Data collection	does not apply
Data analysis	does not apply

For manuscripts utilizing custom algorithms or software that are central to the research but not yet described in published literature, software must be made available to editors/reviewers. We strongly encourage code deposition in a community repository (e.g. GitHub). See the Nature Research guidelines for submitting code & software for further information.

Data

Policy information about availability of data

All manuscripts must include a data availability statement. This statement should provide the following information, where applicable:

- Accession codes, unique identifiers, or web links for publicly available datasets
- A list of figures that have associated raw data
- A description of any restrictions on data availability

The data sets generated during and/or analyzed during the current study are available from the corresponding author on reasonable request.

Field-specific reporting

Please select the one below that is the best fit for your research. If you are not sure, read the appropriate sections before making your selection.

☒ Life sciences ☐ Behavioural & social sciences ☐ Ecological, evolutionary & environmental sciences

For a reference copy of the document with all sections, see nature.com/documents/nr-reporting-summary-flat.pdf

Life sciences study design

All studies must disclose on these points even when the disclosure is negative.

Sample size	3 replicates are commonly accepted for ChIP-qPCR (Figures 1, 2 and 4). Flow cytometry records thousands of independent measurements. Three independent lentiviral CRISPR infections showed comparable results as indicated with error bars (Supplementary Figure 6b, page 33).
Data exclusions	all experiments were included in analysis
Replication	experiments were performed by different individuals at different times
Randomization	does not apply
Blinding	does not apply

Reporting for specific materials, systems and methods

We require information from authors about some types of materials, experimental systems and methods used in many studies. Here, indicate whether each material, system or method listed is relevant to your study. If you are not sure if a list item applies to your research, read the appropriate section before selecting a response.

Materials & experimental systems

n/a	Involved in the study
☐	☒ Antibodies
☐	☒ Eukaryotic cell lines
☒	☐ Palaeontology
☒	☐ Animals and other organisms
☒	☐ Human research participants
☒	☐ Clinical data

Methods

n/a	Involved in the study
☒	☐ ChIP-seq
☐	☒ Flow cytometry
☒	☐ MRI-based neuroimaging

Antibodies

Antibodies used	information is provided in methods section under Chromatin Immunoprecipitation: anti-H3K27me3 (Diagenode, C15410195), anti-Ring1B (Cell Signaling, D22F2), anti-Suz12 (Cell Signaling, D39F6), anti-H3K4me3 (Millipore, 05- 745R), anti-Mel18 (Santa Cruz, sc-10744), anti-Cbx7 (Abcam, ab21873), anti-Rybp (Sigma Aldrich, PRS2227), anti-FLAG (Sigma Aldrich, F1804), anti-H3K27ac (Abcam, ab4729), anti-H2AK119ub (Cell Signaling, D27C4), anti-Gal4 (Santa Cruz, sc-510)
Validation	antibodies were validated by genetic deletion of epitope-encoding gene

Eukaryotic cell lines

Policy information about cell lines

Cell line source(s)	haploid HMSc2 (C57BL/6 3 129 F1)
Authentication	FISH, CNV, SNP, RNA-seq
Mycoplasma contamination	cell lines are regularly tested for mycoplasma infection and are negative
Commonly misidentified lines (See ICLAC register)	does not apply

Flow Cytometry

Plots

Confirm that:

☒ The axis labels state the marker and fluorochrome used (e.g. CD4-FITC).

☒ The axis scales are clearly visible. Include numbers along axes only for bottom left plot of group (a 'group' is an analysis of identical markers).

☐ All plots are contour plots with outliers or pseudocolor plots.

☒ A numerical value for number of cells or percentage (with statistics) is provided.

Methodology

Sample preparation	ES cells are trypsinized and washed prior to preparing single cell suspension for flow analysis either in tubes or 96 well format
Instrument	BD LSR Fortessa
Software	Flowjo software
Cell population abundance	clonal TetO mESCs expressing TetR-Cbx7 or TetR-Rybp were derived by lentivirus infection and subsequent FACS for mCherry-positive cells (~5 - 20%)
Gating strategy	mESCs were gated for shape (Area FSC-Area SSC), doublets (Area FSC-Height FSC)

☐ Tick this box to confirm that a figure exemplifying the gating strategy is provided in the Supplementary Information.
